# Serum Lactate Dehydrogenase Is a Sensitive Predictor of Systemic Complications of Acute Pancreatitis

**DOI:** 10.1155/2022/1131235

**Published:** 2022-10-25

**Authors:** Dong-Ni Huang, Hao-Jie Zhong, Ying-Li Cai, Wen-Rui Xie, Xing-Xiang He

**Affiliations:** ^1^Department of Gastroenterology, The First Affiliated Hospital of Guangdong Pharmaceutical University, Guangzhou, China; ^2^Department of Gastroenterology, Research Center for Engineering Techniques of Microbiota-Targeted Therapies of Guangdong Province, Guangzhou, China; ^3^School of Biology and Biological Engineering, South China University of Technology, Guangzhou, China

## Abstract

**Background:**

Acute pancreatitis (AP) is a common and potentially life-threatening inflammatory disease that can cause various complications, including systemic inflammatory response syndrome (SIRS), pleural effusion, ascitic fluid, myocardial infarction, and acute kidney injury (AKI). However, there is still a lack of rapid and effective indicators to assess the disease. The aim of this study was to investigate the associations of high serum lactate dehydrogenase (LDH) levels with AP severity and systemic complications.

**Methods:**

AP patients treated from July 2014 to December 2020 were retrospectively enrolled. They were divided into elevated (*n* = 93) and normal (*n* = 143) LDH groups. Their demographic data, clinical data, hospital duration, and hospital expenses were analyzed. Linear and binary logistic regression analyses were used to determine whether elevated LDH is a risk factor for AP severity and complications after adjusting for confounders.

**Results:**

There were significant differences in AP severity scores (Ranson, MODS, BISAP, APACHE II, and CTSI), hospital duration, hospital expenses, and the incidences of complications (SIRS, pleural effusion, ascitic fluid, myocardial infarction, and AKI) between the elevated and normal LDH groups. After adjusting for confounders, elevated LDH was associated with AP severity scores and hospital duration and expenses (based on linear regression analyses) and was a risk factor for the occurrence of AP complications and interventions, that is, diuretic and vasoactive agent use (based on binary logistic regression analyses).

**Conclusions:**

Elevated LDH is associated with high AP severity scores and high incidences of complications (SIRS, pleural effusion, ascitic fluid, myocardial infarction, and AKI).

## 1. Introduction

Acute pancreatitis (AP) is the most common pancreatic disease in emergency departments [1]. A prospective study reported that the mortality rate of AP is 9.1% [2]. AP can rapidly cause various local and systemic complications, such as acute kidney injury (AKI), respiratory failure, and heart failure [3, 4], which can lead to death [5–7]. Complications represent the most important factor leading to a poor prognosis in patients with AP. Unfortunately, there is a lack of effective, rapid, and simple markers to assess AP complications.

Lactate dehydrogenase (LDH) has been widely used to evaluate AP severity [8]. In addition, several studies have confirmed that serum LDH is increased in heart and chest diseases (e.g., myocardial infarction [9, 10], acute heart failure [11], and pleural effusion [12]). Moreover, a narrative review indicated that elevated LDH in patients with rhabdomyolysis can predict the occurrence of AKI [13]. However, whether LDH is an indicator of complications in patients with AP remains unclear.

Therefore, this study aims to explore the associations of serum LDH with AP severity and systemic complications.

## 2. Materials and Methods

### 2.1. Participants

Retrospective data on AP patients treated at the First Affiliated Hospital of Guangdong Pharmaceutical University from July 2014 to December 2020 were included. Exclusion criteria were pregnancy, prior organ transplantation, chronic pancreatitis, and incomplete electronic medical records.

### 2.2. Data Collection

All data used for the analysis were extracted from the electronic medical records in the hospital database, including age, gender, alcoholism status, smoking status, complications (systemic inflammatory response syndrome [SIRS], pleural effusion, ascitic fluid, myocardial infarction, pancreatic fluid collection, pancreatic necrosis, AKI, acute respiratory failure, and acute heart failure), interventions (diuretic agent use, vasoactive agent use, tracheal intubation, pancreatic surgery, and transfer to intensive care unit), laboratory data (serum amylase, lipase, and LDH, white blood cells [WBC] count, hypocalcemia [serum calcium <2.12 mmol/L], triglycerides, alanine aminotransferase [ALT], aspartate aminotransferase [AST], serum glucose, and C-reactive protein [CRP]), hospital duration, and hospital expenses.

### 2.3. Definitions

Based on the 2012 revision of the Atlanta classification [4], the diagnosis of AP was based on at least two of the following three features: characteristic abdominal pain, serum amylase or lipase over three times the upper limit of normal, and characteristic findings on abdominal imaging. Elevated serum LDH was defined as >290 U/L [14, 15]. AP severity was assessed according to the Ranson score [16], Multiple Organ Dysfunction Syndrome (MODS) score [17], Bedside Index for Severity in Acute Pancreatitis (BISAP) score [18], Acute Physiology Assessment and Chronic Health Evaluation (APACHE) II score [19], modified Computed Tomography Severity Index (CTSI) [20], and Charlson Comorbidity Index (CCI) [21]. Hypocalcemia was defined as serum calcium <2.12 mmol/L [22]. AKI [23], acute respiratory failure (ARF) [24], and acute heart failure [25] were defined as described in the cited guidelines.

### 2.4. Statistical Analysis

All data analyses were performed in the SPSS software v22.0 (IBM Corporation, Armonk, NY, USA). Data normality was assessed by the Shapiro–Wilk test. Continuous variables with normal and non-normal distribution were analyzed by *t*-tests and Mann–Whitney *U* tests, respectively. Categorical variables were analyzed by chi square tests. Continuous variables are expressed as mean ± standard deviation or median (interquartile range [IQR]), and categorical variables are expressed as frequency (percentage).

Backward stepwise linear regression analyses were used to determine whether elevated LDH (>290 U/L) was a risk factor for AP severity score, hospital duration, and hospital expenses. Backward stepwise binary logistic regression analyses were used to determine whether elevated LDH (>290 U/L) was a risk factor for AP complications and interventions. The analyses were adjusted for potential confounders (gender, age, hypocalcemia, and serum lipase, amylase, glucose, ALT, and AST). Results were considered significant if *P* < 0.05.

### 2.5. Ethical Statement

Ethical approval was granted by the institutional review board of the First Affiliated Hospital of Guangdong Pharmaceutical University, Guangzhou, China (no. 2022-08). The institutional review board waived the requirement for informed consent because of the retrospective nature of the study.

## 3. Results

### 3.1. Patient Characteristics

Based on the inclusion and exclusion criteria, 236 patients with AP were enrolled in this study. They were divided into elevated (*n* = 93) and normal (*n* = 143) LDH groups according to serum LDH levels. The demographic and clinical data of the enrolled patients are presented in Table 1.

### 3.2. AP Severity and Complications

Regarding AP severity, the Ranson, MODS, BISAP, APACHE II, and CTSI scores were significantly higher in the elevated LDH group than those in the normal group (*P* < 0.001; Table 2). The incidence of SIRS was also significantly higher in the elevated LDH group than that in the normal group (*P* < 0.001; Table 2).

Regarding the other AP complications, the incidences of pleural effusion (35.66% vs. 54.84%; *P* = 0.004), ascitic fluid (12.59% vs. 34.41%; *P* < 0.001), myocardial infarction (0.00% vs. 7.53%; *P* = 0.003), pancreatic necrosis (8.40% vs. 18.29%; *P* = 0.032), and AKI (5.59% vs. 20.43%; *P* < 0.001) were significantly higher in the elevated LDH group than those in the normal group (Table 2).

### 3.3. Interventions and Outcomes

Regarding interventions, the rate of diuretic agent use was significantly higher in the elevated LDH group than that in the normal group (26.88% vs. 13.29%; *P* = 0.009; Table 3). Regarding outcomes, hospital duration (9.00; IQR 6.00–14.00 vs. 11.00; IQR 8.00–17.00; *P* = 0.018) and hospital expenses (20676.39; IQR 14305.52–36604.12 vs. 37414.93; IQR 21378.39–60770.88; *P* < 0.001) were significantly higher in the elevated LDH group than those in the normal group (Table 3).

### 3.4. Linear Regression Analyses

The linear regression results showed that after adjusting for potential confounders of the associations of LDH with AP severity scores, the Ranson (*β* = 0.583, standard error [SE] = 0.121; *P* < 0.001), MODS (*β* = 1.126, SE = 0.276; *P* < 0.001), BISAP (*β* = 0.670, SE = 0.147; *P* < 0.001), and APACHE II (*β* = 2.663, SE = 0.715; *P* < 0.001) scores were significantly higher in the elevated LDH group than those in the normal group (Table 4). Hospital duration (*β* = 2.231, SE = 0.915; *P* = 0.016) and hospital expenses (*β* = 19,288.954, SE = 4975.876; *P* < 0.001) were also significantly higher in the elevated LDH group than those in the normal group (Table 4).

### 3.5. Binary Logistic Regression Analyses

The binary logistic regression results showed that after adjusting for potential confounders of the associations of LDH with AP complications and interventions, elevated LDH was a risk factor for pleural effusion (odds ratio [OR] = 1.906; 95% CI [1.057, 3.438]; *P* = 0.032), ascitic fluid (OR = 2.954; 95% CI [1.173–5.736]; *P* = 0.019), SIRS (OR = 3.887; 95% CI [2.049–7.376]; *P* < 0.001), AKI (OR = 4.277; 95% CI [1.656–11.042]; *P* = 0.003), diuretic agent use (OR = 2.516; 95% CI [1.207–5.247]; *P* = 0.014), and vasoactive agent use (OR = 7.285; 95% CI [1.420–37.381]; *P* = 0.017) in patients with AP (Table 5).

## 4. Discussion

In this study, the AP patients with high LDH levels had severer disease (higher Ranson, MODS, BISAP, APACHE II, and CTSI scores), higher risks of complications (SIRS, pleural effusion, ascitic fluid, myocardial infarction, and AKI), and worse prognosis (higher incidence of diuretic agent use, longer hospital duration, and higher hospital expenses) than those with normal LDH levels. Therefore, assessing LDH at the early stage is likely to be useful for predicting the incidence of complications and then improving prognosis by intervening early. To our knowledge, this is the first study to systematically assess the relationship between high LDH levels and multiple complications in AP patients.

Previous studies revealed the close association between LDH and AP severity. First, LDH is one of the 11 metrics in the Ranson score, which is an important milestone regarding AP severity scoring systems [16]. Second, Tian et al. found that the LDH level was approximately two times higher in severe AP patients than that in mild AP patients, and they reported that LDH is a reliable predictor of severe AP (82.7% sensitivity and 96% specificity) [8]. Additionally, a decision tree model [26], which used LDH to predict severe AP, revealed that 63.6% of patients were identified as having severe AP at LDH > 536 U/L, and the overall accuracy of the model was 88.5%. Thus, the decision tree model showed that LDH is likely to be useful for predicting the occurrence of severe AP.

Several recent clinical studies have also indicated that serum LDH is a major risk factor for developing complications in multiple diseases. For instance, LDH levels were significantly elevated in patients with rhabdomyolysis-induced AKI [13]. Second, a study of sickle cell anemia showed a significant positive association between LDH and sickle cell anemia-related leg ulcer recurrence [27]. Third, in cardiothoracic surgery patients, the odds of postoperative cardiopulmonary complications increased by nearly 2-fold for each 100 U/L increase in preoperative LDH [28]. Fourth, a study revealed that LDH was considerably higher in patients with malignant ascites than in patients with benign ascites, and it was a risk factor for malignant ascites [29]. Fifth, a retrospective study of 988 patients with metastatic cancer reported that higher LDH was associated with an increased risk of pleural effusion recurrence [30]. Finally, a recent study reported that patients with higher LDH had a higher risk of stage 3 AKI than patients with lower LDH [31]. In line with these previous studies, we found that the incidences of pleural effusion, ascitic fluid, and AKI were 1.906, 2.594, and 4.277 higher in AP patients with elevated LDH than in those with normal LDH.

The relationship between elevated LDH and AP complications can be partly explained by the following factors. First, several AP complications, such as pleural effusion [32], ascitic fluid [33], and AKI [34], are accompanied by inflammatory reactions, which lead to cell damage and necrosis, cause LDH release into the serum, and ultimately raise the level of LDH. Second, research indicates that the primary source of elevated LDH in severe AP is likely to be the kidneys [35] rather than the pancreas [36], indicating that patients with elevated LDH may have renal complications, such as AKI.

In addition, we found that diuretic agent use was higher in the elevated LDH group than that in the normal group, which may be due to there being a higher incidence of AKI in the elevated LDH group. We also found that AP patients with higher LDH have longer hospital duration and higher hospital expenses, which may be related to severer AP and more complications, thus requiring additional medications and monitoring. Consequently, clinicians should carefully monitor serum LDH in AP patients and provide timely preventative interventions if the LDH level is high, in order to improve prognosis.

Our research has several inevitable limitations. First, the lack of blood samples available in this retrospective study limited research on the mechanisms. Second, although we adjusted for many potential confounders in our analyses, unrecognized residual confounders may still exist, such as the erythrocyte sedimentation rate and the transport time from onset to the hospital. Third, our data came from a single institution, and the sample size was relatively small, so our results require confirmation based on large, multicenter cohort studies.

## 5. Conclusions

Elevated LDH is associated with both high AP severity scores and high incidences of multiple complications (SIRS, pleural effusion, ascitic fluid, and AKI). Therefore, the levels of LDH in AP patients should be closely monitored after hospital admission so that the complications can be recognized early and treated promptly.

## Figures and Tables

**Table 1:** Comparison of demographic and clinical characteristics between AP patients with elevated or normal LDH.

	Normal LDH (*n* = 143)	Elevated LDH (*n* = 93)	*P*-value
Age (years)	59.00 (45.00–79.00)	64.00 (48.00–79.50)	0.761
Male (%)	91 (63.64)	42 (45.16)	0.005
Alcoholism (%)	19 (13.29)	8 (8.60)	0.269
Smoker (%)	35 (24.48)	23 (24.73)	0.964
CCI	1.00 (0.00–2.00)	2.00 (1.00–3.00)	0.050
Hypocalcemia (%)	62 (44.00) (*n* = 141)	59 (65.55) (*n* = 90)	0.003
Amylase (U/L)	296.00 (154.25–718.00) (*n* = 142)	446.00 (189.00–984.00) (*n* = 93)	0.028
Lipase (U/L)	335.30 (95.33–649.25) (*n* = 138)	399.40 (211.15–807.50) (*n* = 93)	0.019
Triglyceride (mmol/L)	1.26 (0.78–2.33) (*n* = 111)	1.31 (0.74–4.35) (*n* = 70)	0.340
WBC count (×10^9^/L)	9.89 (6.70–12.88)	9.85 (6.97–14.20)	0.864
Serum glucose (mmol/L)	6.20 (4.99–8.03) (*n* = 128)	7.88 (5.88–11.03) (*n* = 89)	0.001
ALT (U/L)	33.00 (15.50–92.50) (*n* = 141)	61.00 (20.00–201.50) (*n* = 93)	0.009
AST (U/L)	33.00 (20.00–68.50) (*n* = 141)	73.00 (38.50–346.50) (*n* = 93)	<0.001
CRP (mg/L)	29.80 (11.30–99.05) (*n* = 109)	48.10 (11.75–126.70) (*n* = 77)	0.175

Data are presented as *n* (%) or median (interquartile range). ALT, alanine aminotransferase; AP, acute pancreatitis; AST, aspartate aminotransferase; CRP, C-reactive protein; CCI, Charlson comorbidity index; LDH, lactate dehydrogenase; WBC, white blood cell.

**Table 2:** Comparison of AP severity scores and complications between AP patients with elevated or normal LDH.

	Normal LDH (*n* = 143)	Elevated LDH (*n* = 93)	*P*-value
Severity			
SIRS (%)	31 (21.68)	46 (49.46)	<0.001
Ranson	1.00 (1.00–2.00)	2.00 (2.00–3.00)	<0.001
MODS	0.00 (0.00–2.00)	2.00 (1.00–4.00)	<0.001
BISAP	1.00 (0.00–2.00)	2.00 (1.00–3.00)	<0.001
APACHE II	7.00 (4.00–10.00)	10.00 (6.00–13.00)	<0.001
CTSI	2.00 (2.00–4.00)	4.00 (2.00–6.00)	0.009
Complications			
Pleural effusion (%)	51 (35.66)	51 (54.84)	0.004
Ascitic fluid (%)	18 (12.59)	32 (34.41)	<0.001
Myocardial infarction (%)	0 (0.00)	7 (7.53)	0.003
Pancreatic fluid collection (%)	27 (20.61) (*n* = 131)	26 (31.71) (*n* = 82)	0.068
Pancreatic necrosis (%)	11 (8.40) (*n* = 131)	15 (18.29) (*n* = 82)	0.032
AKI (%)	8 (5.59)	19 (20.43)	<0.001
Acute respiratory failure (%)	6 (4.20)	8 (8.60)	0.161
Acute heart failure (%)	7 (4.90)	8 (8.60)	0.254

Data are presented as *n* (%) or median (interquartile range). AKI, acute kidney injury; AP, acute pancreatitis; APACHE, acute physiologic assessment and chronic health evaluation; BISAP, bedside index for severity in acute pancreatitis; CTSI, computed tomography severity index; LDH, lactate dehydrogenase; MODS, multiple organ dysfunction syndrome; SIRS, systemic inflammatory response syndrome.

**Table 3:** Comparison of interventions and outcomes between AP patients with elevated or normal LDH.

	Normal LDH (*n* = 143)	Elevated LDH (*n* = 93)	*P*-value
Interventions			
Diuretic agent use (%)	19 (13.29)	25 (26.88)	0.009
Vasoactive agent use (%)	3 (2.10)	7 (7.53)	0.091
Tracheal intubation (%)	1 (0.70)	1 (1.08)	1.000
Pancreatic surgery (%)	2 (1.40)	0 (0.00)	0.520
Transfer to intensive care unit (%)	2 (1.40)	3 (3.23)	0.624
Outcomes			
Mortality (%)	2 (1.40)	4 (4.30)	0.337
Hospital duration (days)	9.00 (6.00–14.00)	11.00 (8.00–17.00)	0.018
Hospital expenses (yuan)	20676.39 (14305.52–36604.12)	37414.93 (21378.39–60770.88)	<0.001

Data are presented as *n* (%) or median (interquartile range). AP, acute pancreatitis; LDH, lactate dehydrogenase.

**Table 4:** Linear regression analyses of elevated LDH (>290 U/L) as a risk factor for AP severity scores, hospital duration, and hospital expenses.

	*β*	SE	*P*-value
Ranson	0.583	0.121	<0.001
MODS	1.126	0.276	<0.001
BISAP	0.670	0.147	<0.001
APACHE II	2.663	0.715	<0.001
CTSI	—	—	—
Hospital duration	2.231	0.915	0.016
Hospital expenses	19,288.954	4975.876	<0.001

Analyses were adjusted for gender, age, hypocalcemia, and serum lipase, amylase, glucose, alanine aminotransferase, and aspartate aminotransferase. The normal LDH group served as the reference group. AP, acute pancreatitis; APACHE, acute physiologic assessment and chronic health evaluation; BISAP, bedside index for severity in acute pancreatitis; CTSI computed tomography severity index; MODS, multiple organ dysfunction syndrome.

**Table 5:** Binary logistic regression analyses of elevated LDH (>290 U/L) as a risk factor for AP complications and interventions.

	OR (95% CI)	*P*-value
Complications		
Pleural effusion	1.906 (1.057–3.438)	0.032
Ascitic fluid	2.594 (1.173–5.736)	0.019
Myocardial infarction	143,143,341.1 (0.000–)	0.996
SIRS	3.887 (2.049–7.376)	<0.001
AKI	4.277 (1.656–11.042)	0.003
Acute respiratory failure	—	—
Acute heart failure	3.434 (0.850–13.875)	0.083
Interventions		
Diuretic agent use	2.516 (1.207–5.247)	0.014
Vasoactive agent use	7.285 (1.420–37.381)	0.017
Tracheal intubation	2,709,293,434 (0.000 7.339E+263)	0.942
Pancreatic surgery	—	—
Transfer to intensive care unit	—	—

Analyses were adjusted for gender, age, hypocalcemia, and serum lipase, amylase, glucose, alanine aminotransferase, and aspartate aminotransferase. The normal LDH group served as the reference group. AKI, acute kidney injury; AP, acute pancreatitis; CI, confidence interval; OR, odds ratio; SIRS, systemic inflammatory response syndrome.

## Data Availability

Readers can obtain the datasets of the current study on reasonable request by contacting the author Dong-Ni Huang via email (hdongni@qq.com).
